# *Listeria monocytogenes* Biofilms in the Food Industry: Is the Current Hygiene Program Sufficient to Combat the Persistence of the Pathogen?

**DOI:** 10.3390/microorganisms9010181

**Published:** 2021-01-15

**Authors:** Tina Mazaheri, Brayan R. H. Cervantes-Huamán, Maria Bermúdez-Capdevila, Carolina Ripolles-Avila, José Juan Rodríguez-Jerez

**Affiliations:** Area of Human Nutrition and Food Science, Departament de Ciència Animal i dels Aliments, Facultat de Veterinària, Universitat Autònoma de Barcelona, 08193 Barcelona, Spain; tinamazaheri71@gmail.com (T.M.); brayancervanteshuaman@gmail.com (B.R.H.C.-H.); maria.bermudez@e-campus.uab.cat (M.B.-C.); josejuan.rodriguez@uab.cat (J.J.R.-J.)

**Keywords:** *L. monocytogenes*, food safety, biofilms, cleaning, disinfection

## Abstract

Biofilms contain microbial cells which are protected by a self-produced matrix and they firmly attach themselves to many different food industry surfaces. Due to this protection, microorganisms within biofilms are much more difficult to eradicate and therefore to control than suspended cells. A bacterium that tends to produce these structures and persist in food processing plants is *Listeria monocytogenes*. To this effect, many attempts have been made to develop control strategies to be applied in the food industry, although there seems to be no clear direction on how to manage the risk the bacteria poses. There is no standardized protocol that is applied equally to all food sectors, so the strategies for the control of this pathogen depend on the type of surface, the nature of the product, the conditions of the food industry environment, and indeed the budget. The food industry performs different preventive and corrective measures on possible *L. monocytogenes*-contaminated surfaces. However, a critical evaluation of the sanitization methods applied must be performed to discern whether the treatment can be effective in the long-term. This review will focus on currently used strategies to eliminate biofilms and control their formation in processing facilities in different food sectors (i.e., dairy, meat, fish, chilled vegetables, and ready-to-eat products). The technologies employed for their control will be exemplified and discussed with the objective of understanding how *L. monocytogenes* can be improved through food safety management systems.

## 1. Introduction

Foodborne diseases occur due to the ingestion of food contaminated by biological or chemical agents, consequently causing social, economic, and public health problems [1,2]. In the latest report on the burden of foodborne diseases, it was estimated that 1 in 10 people in the world become ill after ingesting contaminated food and approximately 420,000 people die each year [3], a fact that requires the implementation of rigorous strategic prevention systems, control measures, and surveillance. For all these repercussions, which directly threaten public health and the world economy, it is important to invest in technologies that contribute to preventing foodborne diseases from occurring or to the early detection of threats in terms of food safety [4]. One of the most important prevention tools is the effective application of cleaning and disinfection methods to guarantee food safety [5]. In this regard, there is a high risk of contamination associated with a lack of procedures to ensure surfaces with a zero load of pathogenic microbiota, given the high probability that the product will come into contact with these areas during the handling process [6].

Among the most relevant pathogenic microorganisms at a European level, *Listeria monocytogenes* stands out for its high mortality rate of up to 15.6% [7]. In recent years, a significantly increasing trend in the number of confirmed cases of *L. monocytogenes* in humans has been observed in the EU, up from 1883 confirmed cases in 2013 to 2549 in 2018, representing a notification rate of 0.47 cases per 100,000 inhabitants [7]. Furthermore, Dewey-Mattia et al. [8] indicate the relevance of this pathogen, since it is one of the etiologic agents most involved in hospitalizations and deaths in the USA. Therefore, the control of this pathogen has become one of the targets of greatest interest to the food industry [9].

Biofilms of *L. monocytogenes* on food contact surfaces have been identified as an important pathway for pathogenic persistence and subsequent product contamination [10,11,12]. To this effect, in-depth study of the nature, formation, detection, and elimination of biofilms on surfaces is of great importance due to their impact as a risk factor on outbreaks of foodborne diseases that affect public health [13,14]. The complex nature of biofilms and the capacity of the cells that make them up to strongly fix on surfaces that are difficult to access make the action of the disinfectants that are currently being applied less effective [5]. Another factor to consider is the ability of pathogenic microorganisms to generate resistance to current antimicrobial agents when cells form biofilms [15]. For all these reasons, it must be considered whether the current cleaning and disinfection procedures applied in food industries of different types are effective, or whether new methodologies or strategies are needed to solve the problem.

The objective of the present study was to determine, by means of a literature survey, whether the cleaning and disinfection systems applied nowadays in the food industry are effective for the elimination of *L. monocytogenes* biofilms. To do so, biofilm formation and their involvement in cross-contamination will be discussed to further evaluate the cleaning and disinfection treatments applied in different types of food industries.

## 2. Importance of Cross-Contamination

Over time, inadequate food handling or cooking procedures, breakage of the cold chain, and cross-contamination have been identified as the main drivers of foodborne illnesses [16]. Among these factors, cross-contamination has been highly involved in recent years, accounting for up to 91.7% of cases [17]. Microbial cross-contamination is the transfer of any microorganism from a contaminated biotic or abiotic matrix to an uncontaminated food product [18]. This contamination can happen during any stage of food processing, making the application of control and prevention systems with a global perspective crucial in the food industry [19]. While different raw and processed foods, such as inadequately pasteurized milk and ready-to-eat (RTE) products containing meat, eggs, and fish, have been identified as major sources of *L. monocytogenes* contamination [20], other food products have also been linked with the pathogen due to cross-contamination from industrial surfaces (Table 1). This is related to the ability of *L. monocytogenes* to adhere to and subsequently form biofilms on different food-processing equipment [21,22]. In this regard, equipment such slicing and grinder machines, cutting boards, knives, and tables have been identified as nutritive areas where the pathogen can easily grow due to being difficult to clean and disinfect [23]. Moreover, other industrial surfaces such as floors and sinks have been highlighted as potential focuses of this pathogen and as an initial route point for *L. monocytogenes* transfer to other surfaces [6]. It has been demonstrated that this foodborne pathogen can survive for long periods on industrial surfaces and can be transferred to food products, thus compromising its innocuity [19,24].

Industrial surfaces, then, are important microbial reservoirs that need to be controlled to avoid cross-contamination. There are different determining factors that can influence this phenomenon, such as the fact that when surfaces are dry the risk of cross-contamination occurring is reduced since the growth and survivability of bacteria decreases. However, cross-contamination can be enhanced when surfaces are wet [35]. There are bacteria capable of withstanding prolonged dry conditions on surfaces [36]. Different studies show that there are pathogens that remain viable on dry stainless-steel surfaces for long periods of time, depending on different factors such as the characteristics of a microorganism, the levels of contamination, and its surrounding environment [37,38]. The persistence and resistance of pathogens such as *L. monocytogenes* to extreme environmental conditions is directly related to the ability of microorganisms to form biofilms [39].

## 3. Biofilms

Biofilms are defined as complex microbial communities, irreversibly attached to a biotic or abiotic surface and embedded in an extracellular component matrix (ECM) which exhibits an altered phenotype in relation to the rate of growth and gene transcription [40]. The ability of microorganisms to form biofilms is an adaptive and resistance strategy, which allows them to increase the availability of nutrients for their growth, facilitates the use of water, enables the transfer of genetic material, and what is most worrying for the food industry, gives them resistance to antimicrobial agents [5]. Consequently, routine cleaning and disinfection operations are often ineffective to remove and eliminate the microorganisms that make up these structures [41]. Furthermore, they have been shown to be more resistant to high temperatures, low pH [42], desiccation, UV rays, and salinity, thus increasing their difficulty to be controlled [43]. This resistance facilitates the persistence of the microbial cells that make up the biofilms on food contact surfaces and equipment, constituting a continuous source of contamination [44]. Hence, it is understandable how, according to the National Institute of Health (NIH) and the Center for Disease Control (CDC), biofilms are involved in more than 65% of foodborne diseases. It is therefore important that, to increase their effectiveness, cleaning and disinfection procedures are designed according to the type of problem that is detected.

### 3.1. Initial Attachment and Development

Biofilm formation is a dynamic process that takes place sequentially and includes five main stages. Initial adhesion is the first stage of the biofilm formation process, and is a reversible and weak type of adhesion where planktonic microbial cells adhere to a surface using physical forces and/or appendages such as pili, fimbria, or flagella [5]. The type of surface, the temperature, and the pressure can all modulate this adhesion phenomenon. The electrical charge of the cell surface, the Van der Waals forces, the hydrophobicity of the surface, the steric interactions, and electrostatic are also involved in this process [45]. At this stage, adhesion is reversible until the microorganisms differentiate by triggering morphological changes. Cells can still detach and return to planktonic form when they are in the reversible adhesion phase [46]. Different covalent and hydrophobic interactions occur [47] during irreversible adhesion, the second stage of the biofilm formation process, which is when the cells permanently adhere to each other and the surface [48]. Fixation occurs due to the action of different microbial appendages [49] and by ECM secretion [50]. In the third stage, the simultaneous production of ECM together with the accumulation and growth of attached microorganisms leads to the formation of microcolonies, where the bond between bacteria and the substrate is strengthened and the microbial colony stabilized [49]. Such accumulation stimulates the recruitment of planktonic cells from the surrounding environment through cell-to-cell communication, also named *quorum sensing* [48]. The fourth stage is when there is a mature biofilm due to the development of a highly organized ecosystem and a three-dimensional structure, which can be flat or mushroom-shaped [48]. During maturation, biofilms develop a rigid structure by means of the cellular production of extracellular polymeric substances (EPS) [51]. When using in vitro models to study pathogenic biofilms, it can take between seven [40] and ten days or more [46] to obtain structural maturity, depending on the microorganism and the environmental conditions established. Biofilm maturation is reached when these structures are crossed by water channels or pores, which ensure both the exchange of nutrients and metabolites and eliminate bacterial waste [5].

It has been indicated that when evaluating the effectivity of a cleaning and disinfecting treatment on biofilms it is preferable to use in vitro models that reproduce the structures in this mature stage as this is when they present most resistance and so the results can be more representative of the industrial reality [6,52]. Lastly, the fifth stage is related to dispersion, where sessile cells can return to their planktonic forms and transfer to the environment, once again able to colonize new surfaces [53]. Detachment may be due to low nutrient conditions as a survival mechanism and may be genetically determined. Dispersal is important for microorganisms to escape unfavorable habitats and generate new niches [54].

In the specific case of *L. monocytogenes*, flagella play a predominant role, at least in the early stages of biofilm formation. In this regard, temperature regulates flagellation of *L. monocytogenes* cells [55], so this is a factor that influences the process. The effect of temperature on *L. monocytogenes* biofilm formation has been extensively investigated, demonstrating that the pathogen is flagellated and motile at temperatures ≤30 °C, and generally not flagellated and not motile at temperatures above 30 °C [56]. Although flagellum-mediated attachment is a proven fact of initiation in biofilm formation [57], *L. monocytogenes* can adhere to inert surfaces through a process of passive independent binding of flagella [58]. Tresse et al. [59], have also reported a pH dependence for flagellation of *L. monocytogenes* and its consequences for cell adhesion. It has been shown that not all variables influence biofilm development to the same degree. For example, Poimenidou et al. [60], determined that the impact of nutrient availability on *L. monocytogenes* biofilm formation on stainless steel surfaces is greater than the influence of temperature. Once *L. monocytogenes* is irreversibly attached to the surface its cell mobility and autolytic capacity is reduced, a phenotypic variation that has been indicated to enhance the ability of this opportunistic pathogen to colonize environments [61].

### 3.2. Extracellular Component Matrix (ECM)

ECM is highly hydrated since it incorporates large amounts of water within its structure, reaching up to 97% of the whole biofilm matrix [49]. In most biofilms, the microbial count represents less than 10%, while the matrix can represent more than 90% [62]. The ECM gives biofilms their mechanical stability, mediates their adhesion to surfaces, and forms a cohesive three-dimensional polymeric network that temporarily interconnects and immobilizes biofilm cells [63]. Due to the retention of extracellular enzymes, a versatile external digestive system is generated, sequestering dissolved nutrients from the aqueous phase and allowing them to be used as sources of nutrients and energy [5]. The matrix also acts as a recycling center, keeping all lysed cell components available. This includes DNA, which can represent a gene pool for horizontal gene transfer [63]. ECM can also serve as a source of nutrients, although some components of ECM are slowly biodegradable, and in fact complete degradation of all of their components requires a wide range of enzymes due to their complexity [64]. The matrix generally protects microorganisms against desiccation, oxidizing or charged biocides, some antibiotics, metal cations, and ultraviolet radiation [63]. Among the components that make up the ECM, there are mainly polysaccharides, proteins, and eDNA [13], in addition to various products from bacterial lysis in smaller quantities [65].

Polysaccharides are part of the extracellular matrix and perform various essential functions for the formation of biofilms, generally those associated with adhesion to surfaces and maintenance of structural integrity [66]. Furthermore, the proteins present in the extracellular matrix have functions that allow the growth of the biofilm and the survival of the housed cells through access to nutrients or the regulation of the integrity and stability of the biofilms. Proteins are involved in the formation and stabilization of the matrix polysaccharide network and constitute a link between the bacterial surface and the most glucidic components [67]. Lastly, the eDNA is also an integral part of the biofilm matrix. It acts as an intercellular connector, as a surface adhesive, or even as an antimicrobial, causing cell lysis by chelating lipopolysaccharide stabilizing cations and the bacterial outer membrane [68].

The matrix of *L. monocytogenes* biofilms is mainly composed of proteins [40,69]. In fact, treating biofilms of *L. monocytogenes* with proteases has been shown to damage the development of these structures or to cause cell dispersion [70]. Regarding its content in polysaccharides, Brauge et al. [71], demonstrate that Teichoic acids are the main components of the matrix. Colagiorgi et al. [62] demonstrate that eDNA is a relevant structural component in the *L. monocytogenes* matrix, where it cooperates with polysaccharides and proteins, guaranteeing the integrity of the biofilm [62]. Investigating the composition of these structures in macromolecules is of real importance since knowledge of them leads to the development of new alternative strategies for their elimination.

### 3.3. Mechanisms of Resistance

The resistance acquired by the cells that conform the biofilms is attributed to the properties associated with the biofilm, which include reduced diffusion, physiological changes due to reduced growth rates, and the production of enzymes that degrade antimicrobial substances [47]. It is difficult to establish a single mechanism as the cause of resistance as, in fact, this is given by a combination of many of them. There are studies where it has been observed that disinfectants such as peracetic acid, mercuric chloride, and formaldehyde have been shown to have no effect on certain microorganisms when they are in the form of biofilms [47]. The explanation for the reduced efficacy of these agents against these communities is the incomplete penetration of the biocides through the matrix. It has also been determined that exposure of microorganisms to subinhibitory concentrations of quaternary ammonium compounds (QAC), which can happen when they are in a biofilm form, can lead to the selection of resistant microorganisms that can survive subsequent disinfection treatments applied with higher concentrations of the same compounds [15,72]. To this effect, Chambless et al. [73] propose four possible mechanisms of resistance to biocidal agents of the cells present in the biofilms: (i) difficulty of biocides to penetrate into external areas of the biofilm; (ii) generation of a stress response by some microorganisms; (iii) alteration of the biofilm environment in response; and (iv) microbial resistance by phenotypic differentiation.

In the case of *L. monocytogenes*, it has been determined that the persistence of certain strains, even after cleaning and disinfection, may be related to subinhibitory exposure to disinfectants. This phenomenon can be explained not only by the acquisition of resistance mechanisms by *L. monocytogenes*, but also by the existence of niches or reservoirs in the environment not reached by disinfectants, and by the formation of biofilms and the consequent creation of protected microenvironments [15]. For example, genotypic resistance to QAC by this pathogen has been described. Multiple reflux pumps have been characterized that confer some resistance to QACs. However, as previously commented, the dilution or inactivation of QACs in the environment due to an erroneous cleaning and disinfection protocol also has an influence. This resistance to QAC can end up contributing to its adaptation and environmental persistence [74].

## 4. *Listeria monocytogenes*

### 4.1. Generalities and Characteristics

*L. monocytogenes* is a ubiquitous bacterium which has been isolated from soil, plants, silage, and water, particularly from food processing environments and especially in refrigerated premises, despite them being routinely cleaned and disinfected [75], hence, they are responsible for numerous food outbreaks [76]. The pathogen is also a transitory resident of the intestinal tract in humans, with 2–10% of the general population carrying the microorganism with no apparent health consequences [77]. Its entry into food processing plants can occur through many different routes, from raw materials to contact with contaminated surfaces on equipment or generally in the facilities [23].

*L. monocytogenes* is a rod-shaped, Gram-positive, catalase positive, facultative anaerobic, non-sporulating, psychotrophic mesophilic pathogen [78]. Its ability to survive temperatures between −0.4 to 50 °C, pH from 4.6–9.5, low water activity up to 0.92, and high concentrations of salt (10–2%) and sugar (39.4% sucrose) [79,80], contribute to its persistence in food processing environments, which implies a permanent risk of cross-contamination of products [81].

Up to now, at least 13 distinct *L. monocytogenes* serotypes have been identified, although only serotypes 1/2a, 1/2b, 1/2c and 4b have been involved in 98% of human listeriosis cases worldwide [82]. These 13 serotypes are grouped into 4 different lineages (I, II, III and IV), defined using molecular typing methods such as pulsed field gel electrophoresis (PFGE) [83]. It has been observed that different serotypes can generate different population structures and may have different abilities to combat environmental stress [84]. Regarding incidence, the majority of listeriosis cases are caused by *L. monocytogenes* strains belonging to serotypes 1/2a, 1/2b, and 4b, and to a lesser extent, 1/2c. Interestingly, isolates of serotype 1/2a are highly prevalent in food processing settings, compared to isolates of serotype 4b [85].

### 4.2. Recent Food-Related Crises

*L. monocytogenes* causes a foodborne illness named listeriosis, which primarily affects pregnant women, neonates, the elderly, and immunocompromised individuals [86]. Although *L. monocytogenes* is responsible for only 1% of foodborne illness, its mortality rate is high, far exceeding that of other foodborne pathogens [87]. The first listeriosis food crisis occurred in Canada and was associated with the consumption of contaminated cabbage salad [88]. Since then, there have been various food crises related to this foodborne pathogen, standing out among them the largest listeriosis outbreak ever documented, which occurred in South Africa. Between January 2017 and May 2018, there were 1034 laboratory-confirmed cases of listeriosis, more than 400 (42%) cases in newborns, and 204 associated deaths. The case-fatality rate in South Africa was estimated at 28.6% and was comparable to other reported outbreaks of listeriosis worldwide [89]. In 2018, 2549 confirmed cases of listeriosis in humans were reported in the EU. There has been a statistically significant upward trend in confirmed cases of listeriosis in the EU for the period 2012–2018, with a case fatality in the EU of 15.6% [7]. Focusing on the crises caused by this pathogen in the EU in recent years, in 2018 an outbreak of listeriosis in a Hungarian factory was reported, linked to the production of frozen vegetables and affecting seven countries and a total of 47 people. This factory produced and exported to more than 100 countries [90]. Consequently, European Food Safety Authority (EFSA) recently published an evaluation of the risk posed by *L. monocytogenes* during the processing of frozen fruits and vegetables to enhance its control and avoid subsequent crises [91]. Furthermore, the pathogen has been highly related to processed meat outbreaks [92]. On this regard, in August 2019, the most important listeriosis outbreak in the history of Spain was recorded, affecting over 200 people, of whom three died and five cases resulted in miscarriages [33].

### 4.3. L. monocytogenes and Its Affinity for Materials

*L. monocytogenes* has the ability to adhere to and from biofilms on industrial surfaces, especially where food residues accumulate [93]. As previously discussed, this is a mechanism of potential resistance to antimicrobial agents, biocides, and heat [94]. The resistance of bound bacteria to biocides has been mainly associated with mechanical protection due to the synthesis of ECM and the surrounding nutrients, or with intrinsic physiological factors such as the adaptation of biofilm cells to stresses like acid, oxidative stress, and starvation, among many others [95]. A series of studies on materials commonly used in food facilities and premises have demonstrated the presence of *L. monocytogenes* [23], showing its capacity to adhere to and develop on polystyrene as a material employed to construct drains [96]; polytetrafluoroethylene (PTFE) used in conveyor belts [97]; stainless steel used for the majority of the equipment employed in the food industry [6]; polyester used as a floor sealer [98]; and rubber used in joints and glass [99], or glass and Teflon [100]. However, the degree to which *L. monocytogenes* adheres to these materials differs depending on each type.

## 5. Control Strategies Implemented in the Food Industry

Cleaning and disinfection are an essential part of the Hazard Analysis and Critical Control Points (HACCP) system. Auditing is also an important factor, guaranteeing a reduction in the risk and increasing food safety to provide a safe environment for the manufactured food products in the food industry [101,102]. Sanitation programs have a different objective in food processing environments, among which the following stand out: the removal of visible soil (i.e., organic or inorganic) and allergens, which would be detrimental to the safety or organoleptic quality of subsequent production runs; and the elimination of microorganisms that may cause an alteration of the organoleptic characteristics or can pose a risk to public health [102].

### 5.1. Cleaning

In food processing industries, cleaning is based on the removal of residues and harmful microorganisms such as *L. monocytogenes* to protect food from contamination from surfaces, employing physical or chemical methods [103,104]. Another objective of the cleaning program is to ensure a clean environment for employees, and to prepare equipment and other industrial surfaces in the food area which are difficult to clean, with the aim of extending the product shelf-life and preventing future damage [105,106]. Effective cleaning must break or damage extracellular matrices of biofilms, so that later disinfectants can access the microbial viable cells [107]. Cleaning programs are defined according to the type of dirt present and the type of food processing environment produced. This operation is carried out by applying detergents, which are selected based on the type of product being processed, the type of residue it generates, and the physic-chemical properties of the surfaces being cleaned [108]. Knowing the type of dirt to be removed allows products, systems, and conditions to be chosen to optimize cleaning processes. Cleaning products must have three important characteristics: chelating power, a degreaser, and a dispersant. The first refers to the ability to sequester minerals, preventing them from crystallizing, precipitating, and embedding in the materials on which it is being applied. The second relates to the ability to emulsify and disperse fats, and the third is the ability to break down dirt particles and keep them in suspension [109]. During the cleaning process, a proportion of the microorganisms present can be detached from food contact surfaces. However, some can be non-detached and if water and nutrients are present, during a period of time some microorganisms can adhere to the other surfaces to re-start the cycle of biofilm production. Subsequent disinfection must therefore be applied to remove all foodborne pathogens [110].

### 5.2. Disinfection

Disinfection is the procedure to eliminate the microorganisms completely or to reduce their number to an acceptable level using antimicrobial products, chemical agents, or thermal methodologies [111,112]. This is an important step in the sanitization process as the presence of foodborne pathogens such as *L. monocytogenes* in food industries can be extremely harmful for public health [75,113]. Therefore, *L. monocytogenes* biofilms adhered in processing plants possess increased resistance to environmental conditions, making their removal more difficult [114]. For this reason, selecting the composition of a disinfectant, particularly the active biocidal substance or a combination of several of them, is also dependent on the extracellular matrix component of biofilms. For example, QACs such as benzalkonium chloride are cationic surfactants that act by disrupting lipid membrane bilayers and are effective against a number of pathogenic microorganisms, especially Gram-positive bacteria [115].

### 5.3. Complementary Alternative Strategies

The development of sanitation processes is looking towards alternatives that do not enhance resistance or towards strategies that prevent irreversible adhesion to surfaces and the subsequent development of mature biofilms. The growing negative perception of consumers regarding chemical substances has pushed research towards different environmentally friendly alternatives [116]. Among these are enzymes, bacteriophages, *quorum sensing* inhibitors, essential oils, and others [117]. Enzymatic technology allows for a high degree of personalization in surface sanitization, and so depending on the composition of the biofilm matrix formed by the predominant microorganisms on the surfaces, specific strategies can be defined to optimize their effectiveness. However, its use must be optimized due to its high cost, which is achieved by adjusting the optimal temperature and pH conditions [118]. This allows the concentration of the enzymes to be reduced to a minimum, thus maintaining their effectiveness. In the case of bacteriophages, their antimicrobial action is specific against prokaryotic cells and harmless to humans, animals, and plants [117]. However, other authors disagree on its total safety [119]. The main limitation of phage treatments is their ability to access and attack bacterial cells within the biofilm due to its structure, which acts as a physical obstacle. However, some phages possess depolymerases, which improve the phage invasion and dispersion process through the biofilm under treatment [120]. *Quorum sensing* is a mechanism for regulating gene expression in response to cell population density. In biofilms, it regulates population density and all metabolic activity. This achieves better adaptation to the environment and greater resistance in hostile environments and disinfection processes [121]. Its inhibition is therefore a preventive strategy focused on interrupting biofilm formation by controlling the stages of microbial microcolony formation [122,123]. Essential oils are generally recognized as safe by the US Food and Drug Administration (FDA). Several studies have shown that they have strong antimicrobial and anti-biofilm activity against a variety of microorganisms [124,125,126]. Lastly, another interesting approach to the microbiological control of surfaces is the use of microorganisms that may have the ability to compete with foodborne pathogens and thereby prevent their growth [6]. Even more interesting is the use of microbial species belonging to the resident microbiota of the food industries, which is not only an interesting ecological alternative to explore, but also opens up a field of study with great future prospects [127].

In the specific case of biofilms formed by *L. monocytogenes*, various investigations have been carried out on complementary alternative strategies for their control. It is known that the composition of a biofilm formed by *L. monocytogenes* is mostly protein [62], which is why protease treatments have been used on surfaces and have been shown to trigger an alteration in the development of biofilms [128]. An example of a bacteriophage used is *Listeria* phage P100, which was produced to eliminate biofilms present in processed meat products and on food contact surfaces in processing industries [129]. Gao et al. [130] have investigated the anti-biofilm efficacy of Fingered Citron essential oil (FCEO) against *L. monocytogenes*. In this study, FCEO was found to exhibit strong anti-biofilm activity, inhibiting biofilm formation, eradicating preformed biofilm, and also causing cell death. Further studies are needed to determine the viability of *L. monocytogenes* in industrial conditions.

### 5.4. L. monocytogenes in the Food Industry: Its Control

#### 5.4.1. Dairy Industry

The presence of *L. monocytogenes* in this type of industry is highly relevant given that the pathogen is able to grow at refrigeration temperature and there are certain processing steps that imply the use of low temperatures to preserve raw materials or processed ones, such as when milk is stored in tanks (4 °C). The risk of contaminating dairy products such as cheese by *L. monocytogenes* is directly related to transfer from farm to dairy animals, unhygienic processes, poor pasteurization, and cross-contamination after heat treatment [131]. Within the dairy industry, cheese is considered one of the products most frequently contaminated with *L. monocytogenes* [132]. According to Ramaswamy et al. [133], blue-veined and molded cheeses like Brie, Camembert, Danish Blue, Stilton, and Gorgonzola possess highly nutritive sources and constitute a perfect environment for the growth of this pathogen. Different studies to control *L. monocytogenes* such as Ultra High-Pressure Homogenization (UHPH), pressurized jet water [134], ozone [135], and infrared light [136], have demonstrated that, in Gorgonzola rinds, these technologies are effective in reducing *L. monocytogenes* levels by up to 2–3 log. Treating these type of products with antimicrobials produced by lactic acid bacteria (LAB) or some yeasts such as bacteriocins, ethanol, and other organic acid has been proposed to prevent *L. monocytogenes* growth if cross-contamination of cheeses occurs [137]. In this regard, LAB can be considered a non-chemical alternative in dairy products.

Furthermore, a recent study conducted by Ripolles-Avila et al. [40] shows that *L. monocytogenes* takes seven days to form highly mature biofilms on industrial surfaces when a constant nutrient source is present in the system. This is why a cleaning program is supposed to remove most of the organic matter in food industry areas and the cleaning program should also focus on floors, walls, milking equipment, and difficult to clean areas [138]. A well implemented cleaning program can help to displace milk deposits, dissolve milk proteins, emulsify fat, and aid the removal of dirt. In Guerrero-Navarro et al. [103], two commercial agents were used, one with chemical components and another which was based on a biological solution, the use of enzymes. The results showed that in the dairy industry, enzymatic cleaning agents obtained better results in terms of eliminating organic matter than chemical agents. In this regard, enzymatic products are eco-friendly, therefore not harmful to the environment, and help to reduce wastewater in dairy factories [139].

#### 5.4.2. Meat Processing Industry

In the meat industry, raw meat and RTE products are considered an important vehicle for *L. monocytogenes* transmission [140,141]. In addition to water and handlers, industrial surfaces are an important factor to control and prevent the cross-contamination of meat by *L. monocytogenes* [142]. As has been indicated, for the control of cross-contamination, the most important action is the cleaning and disinfection procedures of industrial surfaces. In this regard, Mazaheri et al. [64] indicate that enzymatic cleaning treatments could remove mature *L. monocytogenes* isolated from stainless steel surfaces in an Iberian pig processing plant with an effectivity of 85–99%. In the same line, Ripolles-Avila et al. [52] compare the effectivity of enzymatic treatment and chlorinated alkaline treatment for the elimination of mature *L. monocytogenes* biofilms from strains also isolated from the meat industry. The results showed a significantly higher effectivity of the enzymatic treatment, demonstrating that the inclusion of new perspectives is needed to combat these structures in the food industry.

Furthermore, some authors have begun to indicate that the total elimination of microorganisms present on industrial surfaces may not always be of interest. For example, in meat products such as certain fermented sausages, it may be desirable that LAB such as *Lactobacillus* spp. and *Leuconostoc* spp. remain on the surfaces to improve and facilitate the fermentation process [74]. In a recent study, Ripolles-Avila et al. [6] observe that the complete elimination or a great reduction of the resident microbiota from the surfaces can enhance the growth of pathogens such as *L. monocytogenes*. This may be because this pathogen is a poor competitor and microorganisms such as LAB or aerobic mesophilic may impede its growth [127]. It may also be due to the production of *L. monocytogenes* inhibitory substances by the resident microbiota. In addition, there are authors who demonstrate an interrelation between microorganisms of different species and pathogens such as *L. monocytogenes*, forming multispecies biofilms [6,143].

#### 5.4.3. Fish Processing Industry

Fish is another of the foods susceptible to *L. monocytogenes* contamination [144]. The microbial contamination of fish and seafood usually happens naturally from the environment during harvesting or occurs during handling and manufacturing in industry [145,146]. Most foodborne pathogens are not able to grow below 10 °C, and the few that do will not grow under 4 °C. Hence the risk of contamination in frozen fish is not extreme [147]. However, different microorganisms such as *Aeromonas* spp., *Plesiomonas* spp., *Clostridium botulinum*, *L. monocytogenes* and *Vibrio* spp. are mainly responsible for fish product spoilage or pathogenicity and can survive at chill temperatures [147,148].

The foodborne pathogen *L. monocytogenes* is ubiquitous and has been found in natural environments such as water or food processing environments, hence it can enter into contact with fish or fish products [74]. Distinct products have been identified as potential sources of *L. monocytogenes* exposure to humans [149]. One of the products most involved is smoked salmon [149]. Research on sanitization in fish factories has been carried out to optimize treatments and increase effectivity. According to Holck et al. [149] and Mcleod et al. [150], UV light could be an alternative for surface decontamination given that it causes microbial inactivation through DNA damage. Chlorine and the products that produce chlorine include hydrogen peroxide and quaternary ammonium compound, two of the disinfectants commonly used in seafood plants [151,152]. A recent study by Lasagabaster et al. [153] proposes the use of bacteriophage as a green strategy to eliminate *L. monocytogenes* biofilms from processing equipment, thus improving seafood safety. Sadekuzzaman et al. [154] explore treating *L. monocytogenes* with bacteriophage reduced biofilm cells on stainless-steel surfaces and rubber surfaces.

#### 5.4.4. Chilled Vegetable Industry

As part of the modern lifestyle, consumers search for healthier, easier to prepare food to reduce preparation time. One of these products is frozen vegetables [155]. As previously indicated, *L. monocytogenes* is a psychotropic bacterium that can grow at refrigeration temperatures and can be present in frozen vegetables [80]. According to EFSA [91], in the EU (2015–2018) there was an outbreak of *L. monocytogenes ST6* related to blanched frozen vegetables in several countries. Evidence of foodborne outbreaks shows that *L. monocytogenes* is the most relevant pathogen associated with this type of product.

The food industry commonly uses chlorine compounds such as chlorhexidine and benzalkonium chloride as disinfectants. However, the resistance of some *L. monocytogenes* isolates to these compounds has been described by Soumet et al. [156]. To this effect, Popowska et al. [157] analyze a total of 96 identified *L. monocytogenes* strains of frozen foods and dairy products, with the aim of determining their susceptibility to benzalkonium chloride and chlorhexidine. In the case of benzalkonium chloride, 16% of the strains were characterized by a reduced susceptibility of 2 to 4 times. For chlorhexidine, however, 82% of the studied strains had a reduced susceptibility to the disinfectant of 2 to 4 times. Furthermore, Godinez-Oviedo et al. [158] characterize *L. monocytogenes* strains isolated from a frozen vegetable processing plant to determine the pathways of contamination of the pathogen. It was determined that the pathogen persistence spaces correspond mainly to those with contact with food, which therefore become an important source of cross contamination.

#### 5.4.5. Ready-to-Eat (RTE) Products Industry

According to previous study by the FDA and the Food Safety and Inspection Service (FSIS) [159] and Pouillot et al. [160], some of the physical and chemical characteristics of RTE such as pH and water activity create a suitable environment for *L. monocytogenes* growth. In this regard, Cossu et al. [161] show that an RTE sandwich factory had a high rate of contamination by *L. monocytogenes*. In this regard, the addition of some of the antimicrobial substances mentioned before could prevent *L. monocytogenes* growth [162]. For example, some combination acidic substances such as sorbic acid and benzenic acid could prevent the growth *L. monocytogenes* in deli-type salads [163,164,165]. The formulation of RTE products including a combination of natural antimicrobial substances has been demonstrated to be effective against *L. monocytogenes* growth during the shelf-life of these products [166].

Control of *L. monocytogenes* in RTE meat products, especially high-risk ones such as hot dogs and deli meats, is based on the use of intensive programs of environmental sanitation, thermal processing such as cooking or pasteurizing in the package, and the incorporation of antimicrobial agents as part of the ingredient formulation (i.e., nitrites, acetates, citrates, diacetates, lactates, and propionates) and sometimes as surface sprays (i.e., lauric arginate and essential oils). The irradiation of RTE products has been widely explored, and the technology has been considered by the FDA to be safe for use in meat and poultry. High hydrostatic pressure has also been evaluated for the control of *L. monocytogenes* in RTE meats with promising safety results, although quality parameters remain compromised. There is also biocontrol, which refers to the use of natural or controlled microorganisms or their antimicrobial products to extend the shelf life or improve the microbiological safety of food. In food, biocontrol is generally carried out by two groups of biological agents: bacteriophages or viruses that specifically infect bacteria and LAB [167]. The Smoked Seafood Working Group (SSWG) has developed guidelines to minimize *L. monocytogenes* contamination of smoked seafood products. The SSWG have identified 5 elements required for a complete *Listeria* spp. control program, namely *Listeria* specific Good Manufacturing Practices and sanitation procedures, employee training, environmental microbiological monitoring and testing, raw material controls, and temperature controls for finished products [168]. Furthermore, Valenzuela-Martinez et al. [169] evaluate the use of vinegar against *L. monocytogenes* in RTE and poultry products stored at 4 °C. The results showed that the vinegar-treated samples resulted in a growth of <1 log CFU/g over the shelf life (120 days). This research provides an alternative to guarantee the food safety of RTE meat products.

## 6. Conclusions

Controlling *L. monocytogenes* at levels that avoid food contamination requires intense efforts, in addition to the application of new technologies as complementary strategies. The most successful hygiene programs for *L. monocytogenes* control are not based on a single type of treatment, whether physical or chemical, but on the combination of the two. Additionally, the inclusion of complementary alternative strategies has been shown to provide interesting results for the control of this pathogen, although more industrial studies are required to verify if they are feasible to apply in industrial environments. In general, the way to control *L. monocytogenes* in food plant environments is to reduce or eliminate the risk of recontamination because of the presence of biofilms in industrial surfaces. Therefore, each and every possible contamination vector must be identified, and control measures implemented to eliminate *L. monocytogenes* at each entry point, including the drains, floors, processing equipment, walls, floors, ceilings, refrigeration units, and air/particle sources. A vision directed at the type of industry in conjunction with the use of conventional and alternative treatments aimed at attacking both the majority residues and the dominant microbiota can help to better control *L. monocytogenes* in the food industry.

## Figures and Tables

**Table 1:** Involvement of the environment and the industrial surfaces on the cross-contamination of food-products by *Listeria monocytogenes*.

Implicated Food Product	Type of Industry	Country	Surface ^x^	Reference
Pasteurized milk cheese	Cheese retailers and cheese processing plant	Canada	Knives, cutting boards, counters, cheese plates, packers, refrigerator handles, brine solution	[25]
Raw and cooked meat of blue crab	Meat processing plants	USA	Floor drain, raw crab cooler, receiving dock, gloves, table	[26]
Whole whitefish, whole salmon and salmon fillet	Smoked fish processing plant	USA	Floors, drains, cutting table, fork truck bars, carts, coolers, trash can, slicer	[26]
Cantaloupe	Cantaloupe farm and processing plant	USA	Cooler, truck, downstream equipment	[27]
Ice cream	Ice cream facilities	USA	Floor, drain	[28]
Pecorino Romano PDO and ricotta salata cheese made from pasteurized or thermized sheep milk	Sheep’s cheese making plants	Italy	Molds, filters, floors, drains, tables, conveyor belts, shelves, washing machines	[29]
Ricotta salata made from pasteurized sheep’s milk	Semi-finished cheeses processing plant	Italy	Washing machine’s brush, manhole, knife, cutting machine, table, floor, trolley shelf	[30]
Raw pork pieces and minced meat samples	Open meat markets	China	Meat mincers, cutting tables and weighing scales.	[31]
Raw pork	Meat retail market	China	Chopping boards and knives, the inner and outer surfaces of chest freezers, meat mincers, hands of people, floors and walls	[32]
Chilled roasted pork meat	Minced meat factory	Spain	Oven cart, larding needles	[33]
Plastic-packaged RTE Meatballs	RTE meat production facility	Germany	Conveyor belts, pulleys, freezers, condensate lines or cable ducts, gullies	[34]

^x^ The type of material that surfaces were made of was included in the search but was not found in the studies analyzed.

## Data Availability

Not applicable.
